# Evaluation of physiological parameters before and after respiratory physiotherapy in newborns with acute viral bronchiolitis

**DOI:** 10.1186/1755-7682-7-3

**Published:** 2014-01-08

**Authors:** Rodrigo A S Gonçalves, Sérgio Feitosa, Cláudia de Castro Selestrin, Vitor E Valenti, Fernando H de Sousa, Arnaldo A F Siqueira, Márcio Petenusso, Luiz Carlos de Abreu

**Affiliations:** 1 Laboratório de Escrita Científica, Departamento de Morfologia e Fisiologia, Faculdade de Medicina do ABC, Av. Príncipe de Gales, 821, 09060-650 Santo André, SP, Brazil; 2 Programa de Pós-Graduação em Fisioterapia, Faculdade de Ciências e Tecnologia, UNESP, Rua Roberto Simonsen, 305, 19060-900 Presidente Prudente, SP, Brazil; 3 Faculdade de Ciências, UNESP, Av. Eng. Luiz Edmundo Carrijo Coube, 14-01, 17033-360 Bauru, SP, Brazil

**Keywords:** Bronchiolitis, Circulatory and Respiratory Physiology, Infant, Newborn, Physiotherapy (Techniques)

## Abstract

**Background:**

Acute viral bronchiolitis is a respiratory disease with high morbidity that affects newborn in the first two years of life. Its treatment with physiotherapy has been highlighted as an important tool, however, there is no consensus regarding its effects on patients improvement. We aimed to evaluate the physiological parameters before and after the procedure respiratory therapy in newborn with acute viral bronchiolitis.

**Method:**

This was a cross sectional observational study in 30 newborns with acute viral bronchiolitis and indicated for physiotherapy care in a hospitalized Urgency and Emergency Unit. It was collected the clinical data of newborn through evaluation form, and we measured heart rate (HR), oxygen saturation (SpO2) and respiratory rate (RR). We measured the variables before physiotherapy treatment, 3, 6 and 9 minutes after the physiotherapy treatment.

**Results:**

There has been no change in HR, however, we observed a decrease in RR at 6 and 9 min compared to 3 min and increase in SpO2 at 3, 6 and 9 min compared to before physiotherapy.

**Conclusion:**

Respiratory physiotherapy may be an effective therapy for the treatment of newborn with Acute Viral Bronchitis.

## Background

The acute viral bronchiolitis (AVB) is a respiratory disease that affects the lower airways through an acute inflammatory process affecting children in the first two years of life and its peak incidence is below 12 months of age. It is of great clinical relevance due to the high morbidity, particularly in the autumn and winter seasons. The mortality of newborns hospitalized with AVB is around 1% [1,2].

Clinically, the AVB is characterized by cough, fever, runny nose, tachypnea, increased respiratory effort and dyspnea. The severity of the symptoms is associated with the inflammatory process caused by viruses, with infiltration of neutrophils, lymphocytes and release of inflammatory mediators that cause edema, muscle spasm and increased mucus production. Chest radiograph shows hyperinflation, atelectasis and peri-bronchial infiltrate [3].

Currently, respiratory physiotherapy (RP) has emerged as an important tool in the treatment of diseases of the respiratory system, acting to increase mucociliary clearance, airway clearance, facilitation of ventilation and gas exchange. The indication of RP in AVB remains controversial with regard to their effects on patients' clinical improvement and reduced hospitalization time. Some authors do not believe in the efficacy of performing RP in infants with AVB [4]. However, other authors found favorable results in physiologic parameters, mucocilicar increased clearance and improved lung auscultation through the application of respiratory therapy protocols [5-7]. Due to the lack of consensus on the issue, we aimed to evaluate the physiological parameters before and after the RP in newborns with AVB.

## Method

We conducted a cross sectional observational study in 30 newborns admitted in the Emergency Unit of São Bernardo do Campo, São Paulo, from October to November, 2008. This study followed all the criteria for human research and was approved by the Research Ethics Committee of the Faculty of Medicine of the ABC Foundation, approval number 258/2008.

### Inclusion criteria

Written consent of the responsible for the patient was signed, possessing prescription of physiotherapy care, clinical and radiological diagnosis of bronchiolitis confirmed, aged between 29 days and 6 months of age and be in the acute phase of the disease.

### Exclusion criteria

Patients using any device of positive pressure ventilation via tracheal tube, nasal prong or face mask, under medication of vasoactive drugs, birth defects, heart disease, neurological disorders or genetic syndromes.

### Variables measured

We measured heart rate (HR) and oxygen saturation (SpO2), which were measured and compiled by a multiparameter monitor Dixtal, DX2020 model. During the pre and post care, physiological variables were recorded, according to protocol formulated by the researchers, who were: HR, measured in whole numbers of heartbeats. Bradycardia was considered when the HR dropped below 80 beats per minute for 20 seconds.

The SpO2 was measured in whole numbers through the pulse oximeter. Hypoxemia was seen when the fall occurred in 10% or more in saturation for more than 20 seconds. No change was made in the fraction of inspired oxygen for 30 minutes before treatment within 30 minutes, to avoid influence on the variation of saturation. The respiratory rate was measured with the aid of a stopwatch Casio brand, model FS-02, followed by quantification, always by the same investigator and in 60 seconds, through the thoraco-abdominal movements.

No control group was used, because each newborn was its own parameter, by comparing the pre and post-therapy. After the initial assessment it was performed the routine physiotherapy care in the Pediatric Nursing team unit, without the intervention of the researchers.

Data were collected by the physiotherapist and researcher obtained from a single meeting in the afternoon, between 2 p.m. and 6 p.m., just before being compiled, and until 9 minutes after the procedure physiotherapy. The completion of the procedures was performed by a physiotherapist care unit, which has not had access to records of data collection. Upon completion of the session, the children underwent a further assessment of physiological parameters for subsequent comparison with previously collected data. The variables were collected at three moments (0–3 minutes, 3–6 minutes, 6–9 minutes).

### Physiotherapy protocol

The neonatal physiotherapy procedures were applied according to the following protocol: measurement of HR, SpO2% and respiratory rate (RR) about 2 minutes before the sessions of physiotherapy and treatment. The protocols included physiotherapy maneuvers reexpansion pulmonary manuals vibration and postural drainage.

### Statistical analysis

At the end of the study, performed the descriptive analysis of qualitative variables, which were presented in terms of their absolute and relative values. Quantitative variables were presented as means and standard deviations. The normality and homogeneity of variances were verified by Kolmogorov-Smirnove and Levene, respectively. To compare the mean values and initial 3, 6, 9 minutes after therapy were statistically different, we used Analysis of Variance (ANOVA) for repeated measures followed by the Friedman post test. In all tests was set at 5% (p <0.05) level for rejection of the null hypothesis, and specific software was used for statistical studies, SPSS 13.0 for Windows.

## Results

The study population consisted of 30 infants aged between 29 days and 6 months old with a mean of 3.4 (± 1.63) months old, of which 66.6% were male. The protocol was applied between the first and seventh day of hospitalization. None of the infants studied were using sedatives or vasoactive drugs, and only three did not use antibiotics. The mean duration of the RP was 18 minutes and 43 seconds (14–20 minutes).

**Figure 1:**
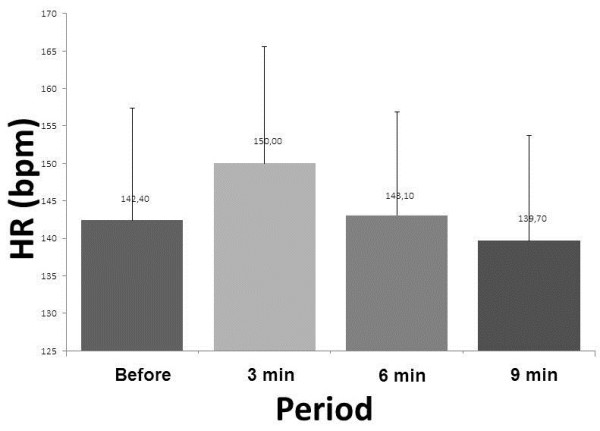
Mean values for heart rate (HR) before treatment and physiotherapy 3, 6 and 9 minutes after the physiotherapy session.

We did not observe significant changes in relation to baseline HR compared the four times (pretreatment vs. Treatment vs 3 min. Treatment vs 6 min. 9 min treatment) (Figure 1).

**Figure 2:**
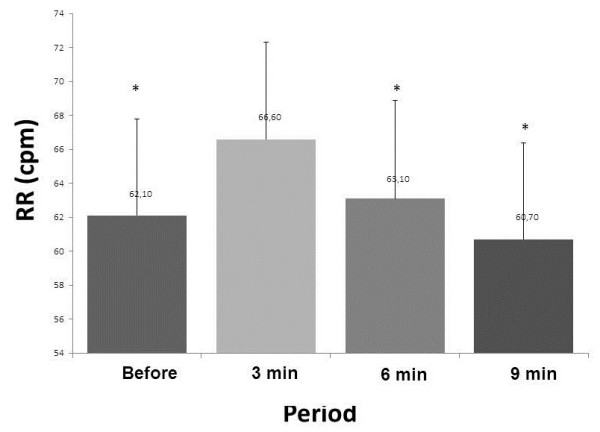
**Mean values for respiratory rate (RR) before treatment and physiotherapy 3, 6 and 9 minutes after the physiotherapy session.** *p <0.05: vs. 3 min.

Regarding RR, there was a statistically significant difference when comparing each of the periods evaluated between the control condition vs. 3 min (p <0.0001), 3 min vs. 6 min (p = 0.000), 3 min vs. 9 min (p = 0.01), demonstrating a period of stabilization (Figure 2).

**Figure 3:**
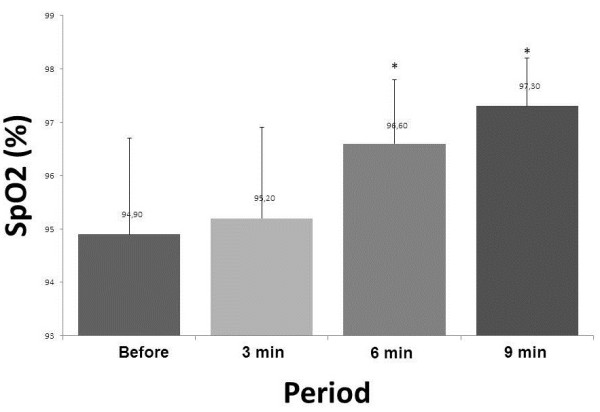
**Mean values for oxygen saturation (SpO2) before treatment and physiotherapy 3, 6 and 9 minutes after the physiotherapy session.** *p <0.05: vs pre.

In relation to the SpO2, it was observed that there was a statistically significant difference between pre and 3 min, pre vs. 6 min and pre vs. 9 min (p <0.0001) (Figure 3).

Table 1 shows the variables presented in the Figures 1, 2 and 3.

**Table 1:** **Mean values for heart rate (HR), respiratory rate (RR) and oxygen saturation (SpO**_**2**_**) before treatment and physiotherapy 3, 6 and 9 minutes after the physiotherapy session**

**Variable**	**Before**	**3 min**	**6 min**	**9 min**
HR (bpm)	142.4 ± 14	150 ± 16	148.1 ± 13	139.7 ± 14
RR (cpm)	62.1 ± 14	66.6 ± 11*	63.1 ± 11**	60.7 ± 12**
SpO_2_ (%)	94.9 ± 1.5	95.2 ± 1.3*	96.6 ± 0.7*	97.3 ± 0.5*

Bpm: Beats per minute; cpm: Cycles per minute). *p <0.05: vs pre; **p <0.05: vs. 3 min.

## Discussion

In this study, we found that physiotherapy procedures in infants with AVB showed no deleterious effects on heart rate and breathing, we observed an increase in the SpO2.

The indication of physical therapy for newborns with AVB is in doubt, it is critical that new research is done because there is no consensus in the literature [8]. French researchers recognize its role as a treatment strategy, prescribed by physicians in 80 to 95% of cases, unlike Anglo-Saxon countries that do not recognize it as part of treatment, due to the lack of controlled studies to prove its effectiveness [1,2].

A review of literature (American Academy of Pediatrics) indicated that the RP did not reduce the length of hospitalization and the need for oxygen and did not improve the clinical severity score for children. Pupin et al. [4], in a study of 81 infants with AVB, in order to compare the effects on physiological parameters in two respiratory therapy protocols, increased expiratory flow (IEF) and vibrocompression associated with postural drainage, decreased RR observed in infants undergoing physiotherapy techniques in relation the control group and the evolution of the HR showed an increase after 10 minutes after the procedure, the group submitted to the IEF protocol followed a decline at the end of the assessment, common to all groups.

An important issue is the fact that the resources used for application of physiotherapy in the pediatric age group were initially adapted from methods used in adult patients, such as tapping, postural drainage and vibration therapy, resources no longer used today because over the years emerged specific techniques appropriate for each age group, consistent with the anatomical and physiological differences of infants [9]. Among them is the increased expiratory flow (IEF) and expiration prolonged slow (ELPr) for use in infants, which were used in this research.

Another study [8] used protocol respiratory therapy in 19 infants with moderate AVB associating ELPr to cough stimulation. The authors observed improvement in symptoms of bronchial obstruction, as well as a decrease in HR and increase in SpO2. The authors stated that there is no risk in implementing this technique and discuss the deleterious results of the application of respiratory therapy in infants with AVB in the literature may be related to protocols that used techniques like tapping and postural drainage.

It has been observed that the performance of respiratory physiotherapy in patients hospitalized for AVB promoted reduction of respiratory distress, eliminating much of secretion aspirated and qualitative improvement auscultation groups who underwent vibration therapy and tapping [10]. The authors believe that reducing respiratory distress is due to increased secretion clearance, which leads to decreased airway resistance and thereby improves the ventilation-perfusion [11,12], since the present study demonstrated improvement of the physiological parameters in the study population following the procedure of physical therapy as noted in the study, against the results obtained with our work.

Using similar method to this study, an investigation evaluated the variability of physiological parameters in 27 preterm infants undergoing physiotherapy and showed a decrease in HR and RR and increased SpO2, suggesting that the reduction in HR and RR leads to decreased energy expenditure because less adenosine triphosphate (ATP) is required to perform the cardiac muscle contractions and bronchial smooth. It is also worth to note that this reduction was accompanied by SpO2 inversely proportional effect [5]. It is also important to mention that physiotherapy protocols in children are different from those applied in adults because it is related to chest expansion [13,14] These results, although applied in different population, corroborate the findings of this research, contributing to the clinical stability of infants.

Our study has some important points to be discussed. The absence of a control group is a limitation of our study, however, this is due to ethical reasons. Another limitation is the short time period assessed that does not allow us to observe how long the beneficial effects are induced. Nonetheless, the advantage of it is the reproducibility of the method, which does not require any change in routine or use of equipment for specific application, it uses the pulse oximeter. We opted for the observation of a single professional in the application of respiratory therapy protocol, in order to avoid technical variations that occur in the application of the maneuvers, because since it is a manual therapy, the human factor is predominant hindering the validation and reliability of the reproducibility of the technique. Studies comparing results of applying the same technique by different professionals are needed to elucidate this issue.

## Conclusions

In this study the application of the protocol of chest physiotherapy in infants with AVB caused no acute effects on beneficial physiological variables of the study population.

## Competing interests

The authors declare that they have no competing interests.

## Authors’ contributions

All authors participated in the acquisition of data and revision of the manuscript. All authors determined the design, interpreted the data and drafted the manuscript. All authors read and gave final approval for the version submitted for publication.

## References

[B1] de Bilderling G Bodart E Bronchiolitis management by the Belgian paediatrician: discrepancies between evidence-based medicine and practice Acta Clin Belg 2003 58 2 98 105 12836492

[B2] Halna M Leblond P Aissi E Dumonceaux A Delepoulle F El Kohe R Impact of the consensus conference on outpatient treatment of infant bronchiolitis. Three-year study in the Nord district of France Presse Med 2005 34 277 281 10.1016/S0755-4982(05)83905-3 15798545

[B3] American Academy of Pediatrics. Subcommittee on Diagnosis and Management of bronchiolitis Diagnosis and management of bronchiolitis Pediatrics 2006 118 1774 1793 17015575 10.1542/peds.2006-2223

[B4] Pupin MK Riccetto AG Ribeiro JD Baracat EC Comparison of the effects that two different respiratory physical therapy techniques have on cardiorespiratory parameters in infants with acute viral bronchiolitis J Bras Pneumol 2009 35 860 867 10.1590/S1806-37132009000900007 19820812

[B5] Selestrin CC Oliveira AG Ferreira C Siqueira AAF Abreu LC Murad N Evaluation of physiological parameters in newborn preterm infants on mechanical ventilation after neonatal physiotherapy procedures J Hum Growth Develop 2007 17 146 155

[B6] de Abreu LC Valenti VE de Oliveira AG Leone C Siqueira AA Herreiro D Wajnsztejn R Manhabusque KV Júnior HM de Mello Monteiro CB Fernandes LL Saldiva PH Chest associated to motor physiotherapy improves cardiovascular variables in newborns with respiratory distress syndrome Int Arch Med 2011 26 4 37 10.1186/1755-7682-4-37 PMC3219586 22029840

[B7] Abreu LC Valenti VE OLIVEIRA AG Leone C Siqueira AAF Gallo PR Fonseca FLA Nascimento VG Saldiva PHN Effects of physiotherapy on hemodynamic variables in newborns with acute respiratory distress syndrome HealthMED Journal 2011 5 528 534

[B8] Postiaux G Dubois R Marchand E Demay M Jacquy J Margiaracina M Effets de la kinésithérapie respiratoire associant expiration lente prolongée et toux provoquée dans la bronchiolite du nourrisson Kinesither Rev 2006 55 35 4

[B9] Bruurs ML van der Giessen LJ Moed H The effectiveness of physiotherapy in patients with asthma: a systematic review of the literature Respir Med 2013 Epub ahead of print 10.1016/j.rmed.2012.12.017 23333065

[B10] Lanza FC Respiratory therapy in infants with bronchiolitis: Perform or not? O Mundo da Saúde São Paulo 2008 2 183 188

[B11] Deschildre A Thumerelle C Bruno B Dubos F Santos C Dumonceaux A Acute bronchiolitis in infants Arch Pediatr 2000 1 21 26 10.1016/s0929-693x(00)88814-4 10793943

[B12] Hough JL Flenady V Johnston L Woodgate PG Chest physiotherapy for reducing respiratory morbidity in infants requiring ventilatory support Cochrane Database Syst Rev 2008 3 CD006445 18646156 10.1002/14651858.CD006445.pub2 PMC12404341

[B13] Mainenti MR de Sousa RC Dias CM Costa SO Ferreira AS de Souza CP Araújo AP Body composition and chest expansion of type II and III spinal muscular atrophy patients J Hum Growth Develop 2013 23 164 169

[B14] Lustosa WA Melo ML Isidório UA de Sousa MA de Abreu LC Valenti VE Cardoso MA de Assis EV Risk factors for recurrent wheezing in infants J Hum Growth Develop 2013 23 203 208

